# Design, Synthesis, Activity and Docking Study of Sorafenib Analogs Bearing Sulfonylurea Unit

**DOI:** 10.3390/molecules201019361

**Published:** 2015-10-23

**Authors:** Chunjiang Wu, Min Wang, Qidong Tang, Rong Luo, Le Chen, Pengwu Zheng, Wufu Zhu

**Affiliations:** 1 School of Pharmacy, Jiangxi Science & Technology Normal University, Nanchang 330013, China; E-Mails: wuchunjiang999@126.com (C.W.); wangmin6029@126.com (M.W.); tangqidongcn@126.com (Q.T.); chenle182@163.com (L.C.); Zhengpw@126.com (P.Z.); 2 Jiangxi Province Institute of Materia Medica, Nanchang 330000, China; E-Mail: 6350173@163.com

**Keywords:** sorafenib, sulfonylurea, VEGFR2/KDR kinase inhibitors, anticancer activity

## Abstract

Two series of novel sorafenib analogs containing a sulfonylurea unit were synthesized and their chemical structures were confirmed by ^1^H-NMR, ^1^^3^C-NMR, MS spectrum and elemental analysis. The synthesized compounds were evaluated for the cytotoxicity against A549, Hela, MCF-7, and PC-3 cancer cell lines. Some of the compounds showed moderate cytotoxic activity, especially compounds 1-(2,4-difluorophenylsulfonyl)-3-(4-(2-(methylcarbamoyl)pyridin-4-yloxy)phenyl)urea (**6c**) and 1-(4-bromophenylsulfonyl)-3-(4-(2-(methylcarbamoyl)pyridin-4-yloxy)phenyl)urea (**6f**) with the IC_50_ values against four cancer cell lines ranging from 16.54 ± 1.22 to 63.92 ± 1.81 μM, respectively. Inhibitory rates against vascular endothelial growth factor receptor-2 (VEGFR2/KDR) kinase at 10 μM of target compounds were further carried out in this paper in order to investigate the target of these compounds. Structure-activity relationships (SARs) and docking studies indicated that the sulfonylurea unit was important to these kinds of compounds. None of the substitutions in the phenoxy group and small halogen atoms such as 2,4-difluoro substitution of the aryl group contributed to the activity. The results suggested that sulfonylurea sorafenib analogs are worthy of further study.

## 1. Introduction

Cancer has been the second biggest killer of human beings, taking the lives of over 7 million people a year [1]. Although a range of antitumor drugs have been discovered in the last decade [2,3,4,5], drug resistance and adverse side effects are still serious problems. Therefore, it remains desirable to develop new antitumor agents with improved tumor selectivity, efficiency, and safety.

**Figure 1:**
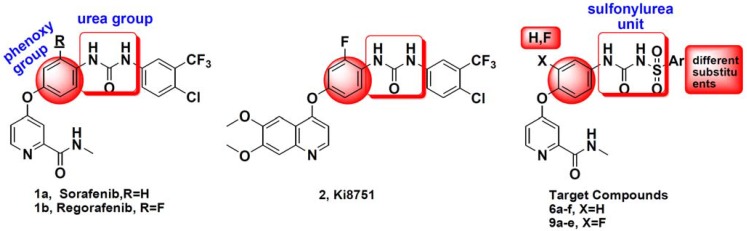
Structures of small-molecule vascular endothelial growth factor (VEGFR2) inhibitors based on the diarylurea scaffold and the target compounds **6a**–**f** and **9a**–**e**.

In recent years, a number of new diarylurea derivatives as VEGFR signal pathway inhibitors have been reported (Figure 1). The main modifications of these compounds were focused on the pyridine ring or phenoxy group. In our previous study, we introduced a sulfonylurea unit instead of urea scaffolds, and the resulting derivatives showed moderate activity as VEGFR2/KDR inhibitors [6].

In order to find compounds with excellent *in vitro*/*in vivo* anti-tumor activity as well as improved pharmacokinetic, further studies on analogous of sulfonylurea-based sorafenib analogs were carried out in this research. Firstly, different substitutions were introduced to the aryl group to investigate the substituents to the activity. Moreover, a fluorine atom was introduced to the phenoxy group, inspired by Regorafenib, KI 8751 (Figure 1) and 6,7-disubstituted-4-phenoxyquinoline derivatives as c-Met kinase inhibitors reported in our previous research [7,8]. The design strategy and structures of the target compounds **6a**–**f** and **9a**–**e** was shown in Figure 1.

Herein, we report the newly synthesized target compounds and their biological activities against four cancer cell lines A549, Hela, MCF-7, PC-3, and VEGFR2/KDR kinase.

## 2. Results and Discussion

**Scheme 1:**
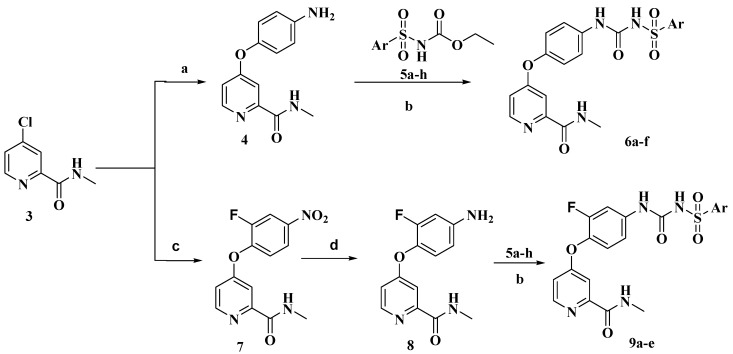
Synthetic route of target compounds **6a**–**f** and **9a**–**e**. *Reagents and conditions*: (**a**) *t*-BuOK, NaI, *N*,*N*-Dimethylformamide (DMF); (**b**) Toluene, reflux, 6 h; (**c**) Chlorobenzene, reflux, 30 h; (**d**) EtOH, FeCl_3_·6H_2_O, N_2_H_4_·H_2_O, reflux, 8 h.

The preparation of target compounds **6a**–**f** and **9a**–**e** is described in Scheme 1.

The synthesis of the key intermediate of 4-(4-aminophenoxy)-*N*-methylpicolinamide **4** was achieved from 4-chloro-*N*-methylpicolinamide **3** as shown in Scheme 1, which has been illustrated in detail in our previous study [6]. Etherification of **3** with 2-fluoro-4-nitrophenol afforded purified **7**, which were reduced using hydrazine hydrate and catalytic amounts of ferric chloride in ethanol to obtain amide **8**. Other key intermediate compounds **5a**–**h** were prepared from commercially available substituted benzene or thiophene via sulfonylation, ammonolysis and acylation reactions according to our previous method [9]. Condensation of **4** or **8** and **6a**–**f** or **9a**–**e** in toluene furnished the target compounds **6a**–**f** and **9a**–**e** in good yield.

### 2.1. Biological Evaluation

Taking sorafenib as reference compound, the target compounds (**6a**–**f** and **9a**–**e**) were evaluated for the cytotoxicity against four cancer cell lines A549 (human lung cancer), Hela (human cervical cancer), MCF-7 (human breast cancer), and PC-3 (human prostate cancer) by 3-(4,5-dimethylthiazolyl-2)-2,5-diphenyltetrazolium bromide (MTT) cell proliferation assay. In addition, these compounds were evaluated for the inhibitory activity against VEGFR2/KDR at 10 μM level *in vitro* by the mobility shift assay together with reference compounds sorafenib and Staurosporine. The results expressed as inhibition rates or IC_50_ values were summarized in Table 1 and the values are the average of at least two independent experiments.

From Table 1, we could see that six target compounds showed moderate cytotoxicity against A549, Hela, MCF-7, and PC-3 cancer cell lines. Among them, compounds **6c** and **6f** showed better activity with the IC_50_ values against four cancer cell lines ranging from 16.54 μM to 63.92 μM, respectively. Moreover, compound **6f** showed good activity against all four cancer cell lines. Generally, the first series compounds (**6a**–**f**) had more potent activities to cancer cells than the second series compounds (**9a**–**e**) which indicated that the change in the substitution of the phenoxy group from H to F decrease the cytotoxicity of the sulfonylurea derivatives. Different substitutions of the aryl group affected the cytotoxicity of target compounds. Small halogen atoms (2,4-di F (**6c**) or 4-Br (**6f**)) substitutions of the aryl group contributed to the activity of the first series, while there is no significant regularity of the second series.

**Table 1:** Structures and activity of target compounds **6a**–**f** and **9a**–**e**.

Compounds No.	Ar	VEGFR2/KDR Inhibitory Rate@10μM (%)	IC_50_(μM) ^a^			
			A549	Hela	MCF-7	PC-3
**6a**	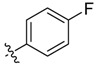	23.6% ± 12.9%	>100	>100	>100	>100
**6b**	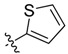	54.0% ± 2.7%	65.86 ± 2.01	>100	72.43 ± 1.96	>100
**6c**	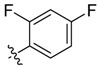	75.8% ± 5.5%	27.04 ± 1.43	>100	>100	25.35 ± 1.73
**6d**	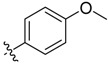	61.3% ± 9.6%	86.91 ± 2.03	>100	80.56 ± 2.04	>100
**6e**	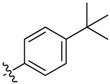	31.4% ± 7.2%	>100	>100	>100	68.87 ± 2.14
**6f**	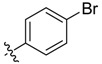	46.6% ± 1.8%	32.59 ± 1.51	63.92 ± 1.81	16.54 ± 1.22	17.97 ± 1.56
**9a**	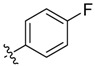	<20.0%	>100	42.43 ± 1.93	17.19 ± 1.54	ND ^c^
**9b**	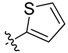	<20.0%	>100	>100	>100	ND
**9c**	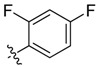	<20.0%	57.42 ± 1.89	>100	>100	ND
**9d**	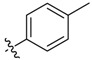	<20.0%	33.22 ± 1.82	24.65 ± 1.69	>100	ND
**9e**	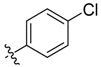	<20.0%	>100	44.32 ± 1.75	69.25 ± 1.96	ND
**Sorafenib ^b^**	-	94.9% ± 1.1%	6.53 ± 0.82	8.08 ± 0.91	4.21 ± 0.62	11.05 ± 1.07
**Staurosporine ^b^**	-	97.3% ± 2.82%	ND	ND	ND	ND

^a^ The values are an average of two separate determinations; ^b^ Used as a positive controls; ^c^ ND: Not determined.

Inhibitory rates against VEGFR2/KDR kinase at 10 μM of target compounds were further carried out in this paper. Compounds **6b**–**d**, **6f** exhibit moderate inhibitory rates against VEGFR2/KDR kinase at 10 μM. Compound **6c** showed the best results, with the inhibitory rate of 75.8% ± 5.5%. It is also suggested that small halogen atoms benefit the activity of target compounds.

Although all of the target compounds showed less activity than the positive compounds sorafenib and staurosporine, it still showed that the replacement of urea with sulfonylurea unit is important to the activity of this series of compounds. More compounds of sorafenib analogs bearing a sulfonylurea may be screened by replacing the aryl groups with heterocyclic rings in further study.

### 2.2. Molecular Docking Study

To explore the binding modes of target compounds with VEGFR2, molecular docking simulation studies were carried out by using SURFLEX-DOCK module of SYBYL package version. The co-crystal structure of sorafenib with VEGFR2/KDR kinase was selected as the docking model (PDB ID code: 4ASD [10]). Based on the *in vitro* inhibition results, we selected the best VEGFR2 inhibitor **6c** as a ligand example.

**Figure 2:**
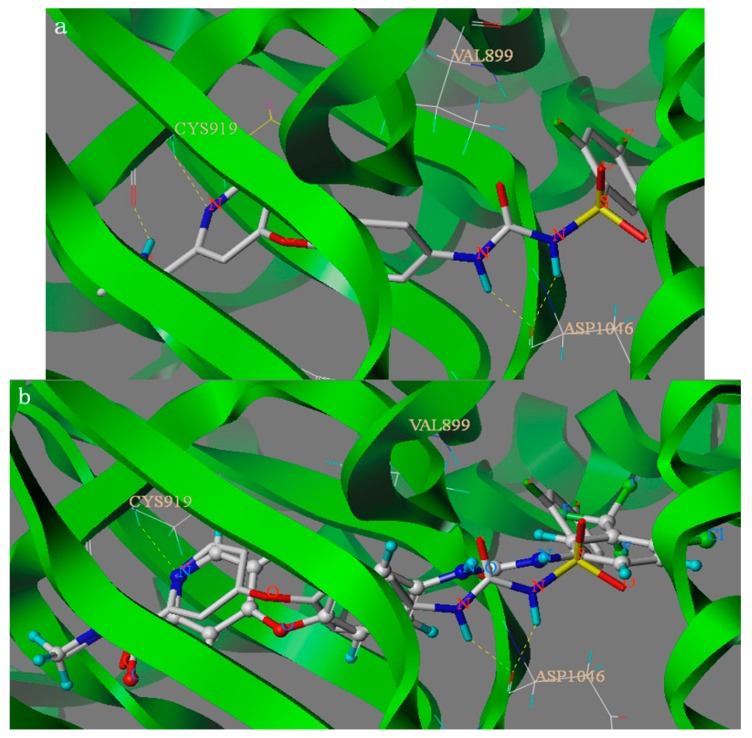
Binding models of compound **6c** ((**a**) shown in Capped Sticks) and parent compound Sorafenib ((**b**) shown in Ball and Stick) target into the active site of VEGFR2. Hydrogen bonds were showed in dashed lines (yellow).

The binding modes of compound **6c** and lead compound were shown in Figure 2 and the docking score of compound **6c** and lead compound were 9.148 and 10.447. As depicted in Figure 2, compound **6c** and Sorafenib can nearly overlap in the binding model and amide group and urea group formed four hydrogen bonds with residues CYS919 and ASP1046, respectively. The H-bond distances are 1.66 Å, 1.71 Å, 1.92 Å and 2.01 Å, respectively. The results showed that the four hydrogen bonds can be combined with VEGFR protein residues. Analysis of compound **6c**’s binding mode in the active binding site demonstrated that the docking mode of the **6c** is similar to the lead compound sorafenib with the same H-bond between amide group and residues CYS919. The four hydrogen bonds play an important role in increasing the inhibitory potency of sulfonylurea derivatives against VEGFR2/KDR kinase according to the docking results and the activity. However, from the docking score of compound **6c** and lead compound, we could see why the activity of compound **6c** was lower than lead compound. Furthermore, the docking results also give us a new direction to design new VEGFR2/KDR inhibitors that can interact with CYS919 and ASP1046. The above-mentioned results of SAR analysis and molecular docking study may allow the rational design of more potent VEGFR2/KDR inhibitors.

## 3. Experimental Section

### 3.1. Chemistry

All melting points were obtained on a Büchi Melting Point B-540 apparatus (Büchi Labortechnik, Flawil, Switzerland) and were uncorrected. NMR spectra were performed using Bruker 400 MHz spectrometers (Bruker Bioscience, Billerica, MA, USA) with TMS as an internal standard. Mass spectra (MS) were taken in ESI mode on Agilent 1100 LC-MS (Agilent, Palo Alto, CA, USA). All the materials were obtained from commercial suppliers and used without purification, unless otherwise specified. Yields were not optimized. TLC analysis was carried out on silica gel plates GF254 (Qindao Haiyang Chemical, Qingdao, China). Elemental analysis was determined on a Carlo-Erba1106 Elemental analysis instrument (Carlo Erba, Milan, Italy).

General Procedure for Preparation of Compounds **5a**–**h**.

Compounds **5a**–**h** were synthesized according to the reported procedures [9,11,12].

*4-(4-Aminophenoxy)-N-methylpicolinamide* (**4**)

The synthesis of the key intermediates of 4-(4-aminophenoxy)-*N*-methylpicolinamide **4** has been illustrated in detail in our previous study [6].

*4-(2-Fluoro-4-nitrophenoxy)-N-methylpicolinamide* (**7**)

A stirring mixture of an appropriate 4-chloro-*N*-methylpicolinamide (0.12 mol) and 2-fluoro-4-nitrophenol (0.18 mol) in chlorobenzene (200 mL, 5 *v*/*w*) was refluxed for about 30 h. The resulting mixture was cooled, and filtered. The filter cake was washed with saturated K_2_CO_3_ solution, and recrystallized with ethanol to give the corresponding nitro compound **7**.

*4-(4-Amino-2-fluorophenoxy)-N-methylpicolinamide* (**8**)

To a refluxing solution of an appropriate nitro compound (0.1 mol), FeCl_3_·6H_2_O and the activated carbon in EtOH (200 mL, 10 *v*/*w*) was added hydrazine hydrate in batches. The mixture was kept at this temperature for more 8 h. After completion of the reaction as indicated by TLC, the mixture was filtered immediately, and the filtrate was cooled, filtered to obtain the corresponding aniline **8**.

General Procedure for Preparation of Compounds **6a**–**f** and **9a**–**e**.

4-(4-Aminophenoxy)-*N*-methyl-pyridine carboxamide (0.41 mmol) or 4-(4-amino-2-fluorophenoxy)-*N*-methylpicolinamide and compounds **5a**–**h** (0.41 mmol) was added to the toluene (5 mL). Refluxed for about 6 h to precipitate a brown solid, filtration and drying gave the title compounds **6a**–**f** and **9a**–**e**.

Compound **6a**. Yield: 40.0%. ESI-MS [M + H]^+^ *m*/*z*: 443.1; m.p.: 187–190 °C; ^1^H-NMR (400 MHz, DMSO) δ 9.09 (s, 1H), 8.77 (s, 1H), 8.49 (d, *J* = 5.0 Hz, 1H), 8.09–8.00 (m, 2H), 7.47 (d, *J* = 8.9 Hz, 3H), 7.34 (s, 1H), 7.15 (s, 1H), 7.13 (s, 1H), 7.12–7.08 (m, 1H), 2.77 (d, *J* = 4.1 Hz, 3H). ^13^C-NMR (100 MHz, DMSO) δ 166.2(C), 164.3(C), 163.9(C), 152.9(C), 150.8(C), 150.0(C), 149.12(CH), 136.8(C), 136.2(C), 131.2(CH), 131.1(CH), 121.8(2CH), 121.5(2CH), 116.5(CH), 116.0(CH), 114.4(CH), 109.3(CH), 26.42(CH_3_). Anal. calcd. for C_20_H_17_FN_4_O_5_S (%): C, 54.05; H, 3.86; N, 12.61. Found (%): C, 54.01; H, 3.83; N, 12.57.

Compound **6b**. Yield: 45.4%. ESI-MS [M + H]^+^ *m*/*z*: 431.1; m.p.: 117–121 °C; ^1^H-NMR (400 MHz, DMSO) δ 9.07 (s, 1H), 8.79 (d, *J* = 4.3 Hz, 1H), 8.49 (d, *J* = 5.6 Hz, 1H), 8.02 (dd, *J* = 5.0, 1.3 Hz, 1H), 7.80 (dd, *J* = 3.7, 1.3 Hz, 1H), 7.50 (d, *J* = 9.0 Hz, 2H), 7.35 (d, *J* = 2.4 Hz, 1H), 7.24–7.19 (m, 1H), 7.17 (s, 1H), 7.15 (s, 1H), 7.12 (dd, *J* = 5.6, 2.6 Hz, 1H), 2.77 (d, *J* = 4.8 Hz, 3H). Anal. calcd. for C_18_H_16_N_4_O_5_S_2_ (%): C, 49.99; H, 3.73; N, 12.95. Found (%): C, 49.96; H, 3.71; N, 12.93.

Compound **6c**. Yield: 33.6%. ESI-MS [M + H]^+^ *m*/*z*: 461.1; m.p.: 236–237 °C; ^1^H-NMR (400 MHz, DMSO) δ 8.90 (s, 1H), 8.80 (d, *J* = 4.6 Hz, 1H), 8.51 (d, *J* = 5.5 Hz, 1H), 7.60 (d, *J* = 8.8 Hz, 2H), 7.38 (d, *J* = 2.2 Hz, 1H), 7.19 (s, 1H), 7.17 (s, 1H), 7.15 (d, *J* = 2.5 Hz, 1H), 2.79 (d, *J* = 4.7 Hz, 3H). Anal. calcd. for C_20_H_16_F_2_N_4_O_5_S (%): C, 51.95; H, 3.49; N, 12.12. Found (%): C, 51.92; H, 3.46; N, 12.08.

Compound **6d**. Yield: 41.7%. ESI-MS [M + H]^+^ *m*/*z*: 455.2; m.p.: 100–104 °C; ^1^H-NMR (400 MHz, DMSO) δ 8.97 (s, 1H), 8.78 (s, 1H), 8.49 (d, *J* = 5.6 Hz, 1H), 7.90 (d, *J* = 8.9 Hz, 2H), 7.46 (d, *J* = 8.9 Hz, 2H), 7.34 (d, *J* = 2.2 Hz, 1H), 7.15 (s, 2H), 7.13 (s, 2H), 7.10 (d, *J* = 2.6 Hz, 1H), 3.85 (s, 3H), 2.78 (d, *J* = 4.7 Hz, 3H). Anal. calcd. for C_21_H_20_N_4_O_6_S (%): C, 55.26; H, 4.42; N, 12.27. Found (%): C, 55.21; H, 4.39; N,12.23.

Compound **6e**. Yield: 32.8%. ESI-MS [M + H]^+^ *m*/*z*: 481.2; m.p.: 65–69 °C; ^1^H-NMR (400 MHz, DMSO) δ 9.08 (s, 1H), 8.80 (d, *J* = 4.5 Hz, 1H), 8.49 (d, *J* = 5.6 Hz, 1H), 7.91 (d, *J* = 8.4 Hz, 2H), 7.76 (d, *J* = 8.3 Hz, 3H), 7.48 (d, *J* = 8.8 Hz, 2H), 7.36 (s, 1H), 7.16 (s, 1H), 7.14 (s, 1H), 7.12 (d, *J* = 5.7 Hz, 1H), 2.78 (d, *J* = 4.7 Hz, 3H), 1.30 (s, 9H). Anal. calcd. for C_24_H_26_N_4_O_5_S (%): C, 59.74; H, 5.43; N, 11.61. Found (%): C, 59.71; H, 5.39; N, 11.58.

Compound **6f**. Yield: 39.2%. ESI-MS [M + H]^+^ *m*/*z*: 505.0; m.p.: 119–122 °C; ^1^H-NMR (400 MHz, DMSO) δ 9.12 (s, 1H), 8.76 (s, 1H), 8.48 (s, 1H), 7.87–7.77 (m, 4H), 7.47 (s, 2H), 7.33 (s, 1H), 7.13 (s, 3H), 2.76 (s, 3H). Anal. calcd. for C_20_H_17_BrN_4_O_5_S (%): C, 47.53; H, 3.39; N, 11.09. Found (%): C, 47.51; H, 3.36; N, 11.05.

Compound **9a**. Yield: 42.4%. ESI-MS [M + H]^+^ *m*/*z*: 463.1; m.p.: 186.5–187.7 °C; ^1^H-NMR (400 MHz, DMSO) δ 8.68 (s, 1H), 8.45–8.38 (m, 1H), 7.96 (s, 1H), 7.79 (d, *J* = 5.4 Hz, 1H), 7.46 (d, *J* = 13.1 Hz, 1H), 7.39 (t, *J* = 8.7 Hz, 1H), 7.33 (d, *J* = 9.9 Hz, 2H), 7.06 (d, *J* = 8.8 Hz, 1H), 6.94 (t, *J* = 8.9 Hz, 1H), 6.45 (d, *J* = 13.1 Hz, 1H), 6.37 (d, *J* = 8.3 Hz, 1H), 2.70 (d, *J* = 3.3 Hz, 3H). Anal. calcd. for C_20_H_16_F_2_N_4_O_5_S (%): C, 51.95; H, 3.49; N, 12.12. Found (%): C, 51.92; H, 3.44; N, 12.09.

Compound **9b**. Yield: 43.7%. ESI-MS [M + H]^+^ *m*/*z*: 451.2; m.p.: 182.5–183.6 °C; ^1^H-NMR (400 MHz, DMSO) δ 8.68 (s, 1H), 8.46–8.36 (m, 1H), 7.74 (s, 1H), 7.56 (s, 1H), 7.47 (s, 1H), 7.27 (s, 1H), 7.21–7.09 (m, 1H), 7.05 (s, 1H), 6.93 (d, *J* = 8.5 Hz, 1H), 6.46 (d, *J* = 13.2 Hz, 1H), 6.37 (d, *J* = 7.9 Hz, 1H), 2.70 (s, 3H). ^13^C-NMR (100 MHz, DMSO) δ 166.6(C), 165.7(C), 164.1(C), 155.1(C), 150.9(C), 140.7(CH), 137.8(C), 135.4(C), 134.6(CH), 134.3(CH), 130.5(C), 127.7(CH), 124.4(CH), 116.5(CH), 113.6(CH), 110.8(CH), 108.6(CH), 26.4(CH_3_). Anal. calcd. for C_18_H_15_FN_4_O_5_S_2_ (%): C, 47.99; H, 3.36; N, 12.44. Found (%): C, 47.95; H, 3.31; N, 12.40.

Compound **9c**. Yield: 31.5%. ESI-MS [M + H]^+^ *m*/*z*: 481.2; m.p.: 126.7–128.3 °C; ^1^H-NMR (400 MHz, DMSO) δ 8.76 (s, 1H), 8.53–8.46 (m, 1H), 7.86 (dd, *J* = 14.9, 8.5 Hz, 1H), 7.71 (s, 1H), 7.52 (dd, *J* = 20.7, 9.9 Hz, 1H), 7.34 (d, *J* = 2.3 Hz, 1H), 7.25 (s, 1H), 7.17 (d, *J* = 8.2 Hz, 1H), 7.14–7.10 (m, 1H), 7.02 (t, *J* = 9.0 Hz, 1H), 6.54 (d, *J* = 13.2 Hz, 1H), 6.45 (d, *J* = 8.4 Hz, 1H), 2.78 (d, *J* = 3.8 Hz, 3H). Anal. calcd. for C_20_H_15_F_3_N_4_O_5_S (%): C, 50.00; H, 3.15; N, 11.66. Found (%): C, 49.96; H, 3.12; N, 11.63.

Compound **9d**. Yield: 45.3%. ESI-MS [M + H]^+^ *m*/*z*: 459.2; m.p.:93.5–95.6 °C; ^1^H-NMR (400 MHz, DMSO) δ 8.68 (s, 1H), 8.40 (d, *J* = 5.3 Hz, 1H), 7.77 (d, *J* = 7.4 Hz, 1H), 7.62 (d, *J* = 7.6 Hz, 1H), 7.35 (d, *J* = 8.1 Hz, 1H), 7.28 (d, *J* = 8.1 Hz, 3H), 7.18 (s, 1H), 7.05 (s, 1H), 6.94 (t, *J* = 9.0 Hz, 1H), 6.45 (d, *J* = 13.0 Hz, 1H), 6.36 (d, *J* = 8.1 Hz, 1H), 2.70 (s, 3H), 2.41 (s, 3H). ^13^C-NMR (100 MHz, DMSO) δ 166.6(C), 165.7(C), 164.1(C), 153.1(C), 150.9(C), 144.4(CH), 142.4(C), 137.9(C), 137.8(C), 137.5(C), 129.9(CH), 129.1(CH), 127.9(CH), 126.1(CH), 124.4(CH), 116.4(CH), 113.7(CH), 110.7(CH), 108.5(CH), 26.4(CH_3_), 21.5(CH_3_). Anal. calcd. for C_21_H_19_FN_4_O_5_S (%): C, 55.02; H, 4.18; N, 12.22. Found (%): C, 54.98; H, 4.14; N, 12.18.

Compound **9e**. Yield: 44.6%. ESI-MS [M + H]^+^ *m*/*z*: 479.2; m.p.: 118.5–120.2 °C; ^1^H-NMR (400 MHz, DMSO) δ 8.68 (s, 1H), 8.40 (d, *J* = 5.4 Hz, 1H), 7.78 (dd, *J* = 15.1, 8.7 Hz, 1H), 7.63 (s, 1H), 7.47 (d, *J* = 12.1 Hz, 1H), 7.41 (d, *J* = 10.2 Hz, 1H), 7.27 (s, 1H), 7.08 (d, *J* = 7.4 Hz, 2H), 7.05 (d, *J* = 5.6 Hz, 1H), 6.94 (t, *J* = 9.0 Hz, 1H), 6.46 (d, *J* = 13.2 Hz, 1H), 6.37 (d, *J* = 8.6 Hz, 1H), 2.70 (d, *J* = 3.5 Hz, 3H). ^13^C-NMR (100 MHz, DMSO) δ 166.6(C), 165.7(C), 164.1(C), 155.1(C), 150.9(C), 149.9(CH), 137.7(C), 137.6(C), 135.5(C), 133.7(C), 133.6(C), 129.3(CH), 128.6(CH), 125.7(CH), 124.3(CH), 116.6(CH), 113.6(CH), 110.9(CH), 108.5(CH), 26.4(CH_3_). Anal. calcd. for C_20_H_16_ClFN_4_O_5_S (%): C, 50.16; H, 3.37; N, 11.70. Found (%): C, 50.12; H, 3.34; N, 11.66.

### 3.2. VEGFR2/KDR Kinase Assay

The inhibitory activity against VEGFR2/KDR at 10 μM level *in vitro* was evaluated through the mobility shift assay together with reference compounds sorafenib and Staurosporine[13]. All kinase assays were performed in 96-well plates in a 50 μL reaction volume. The kinase buffer contains 50 mM HEPES, pH 7.5, 10 mM MgCl_2_, 0.0015% Brij-35 and 2 mM DTT. The stop buffer contains 100 mM HEPES, pH 7.5, 0.015% Brij-35, 0.2% Coating Reagent #3 and 50 mM EDTA. Dilute the compounds to 500 μM by 100% DMSO, then transfer 10 μL of compound to a new 96-well plate as the intermediate plate, add 90 μL kinase buffer to each well. Transfer 5 μL of each well of the intermediate plate to 384-well plates. The following amounts of enzyme and substrate were used per well: kinase base buffer, FAM-labeled peptide, ATP and enzyme solution. Wells containing the substrate, enzyme, DMSO without compound were used as DMSO control. Wells containing just the substrate without enzyme were used as low control. Incubate at room temperature for 10 min. Add 10 μL peptide solution to each well. Incubate at 28 °C for specified period of time and stop reaction by 25 μL stop buffer. At last collect data on Caliper program and convert conversion values to inhibition values. Percent inhibition = (max − conversion)/(max − min) × 100. “max” stands for DMSO control; “min” stands for low control.

### 3.3. Cytotoxicity Assay in Vitro

The cytotoxic activities of compounds were evaluated with A549, Hela, MCF-7 and PC-3 cell lines by the standard MTT assay *in vitro*, with compounds VEGFR inhibitor Sorafenib as positive control. The cancer cell lines were cultured in minimum essential medium (MEM) supplement with 10% fetal bovine serum (FBS). Approximately 4 × 10^3^ cells, suspended in MEM medium, were plated onto each well of a 96-well plate and incubated in 5% CO_2_ at 37 °C for 24 h. The test compounds at indicated final concentrations were added to the culture medium and the cell cultures were continued for 72 h. Fresh MTT was added to each well at a terminal concentration of 5 mg/mL and incubated with cells at 37 °C for 4 h. The formazan crystals were dissolved in 100 μL DMSO each well, and the absorbency at 492 nm (for absorbance of MTT formazan) and 630 nm (for the reference wavelength) was measured with the ELISA reader. All of the compounds were tested three times in each of the cell lines. The results expressed as inhibition rates or IC_50_ (half-maximal inhibitory concentration) were the averages of two determinations and calculated by using the Bacus Laboratories Incorporated Slide Scanner (Bliss) software (the Bacus Laboratories Inc. Slide Scanner (BLISS) system, Lombard, IL, USA).

### 3.4. Docking Studies

To further elucidate the binding mode of compounds, docking analysis was performed. The three-dimensional structure of the VEGFR2 (PDB code: 4ASD) were obtained from RCSB Protein Data Bank [14]. Hydrogen atoms were added to the structure allowing for appropriate ionization at physiological pH. The protonated state of several important residues, such as CYS919, and ASP1046 were adjusted by using SYBYL6.9.1 (Tripos, St. Louis, MO, USA) in favor of forming reasonable hydrogen bond with the ligand. Molecular docking analysis was carried out by the SURFLEX-DOCK module of SYBYL 6.9.1 package (Tripos) to explore the binding model for the active site of VEGFR2 with its ligand. All atoms located within the range of 5.0 Å from any atom of the cofactor were selected into the active site, and the corresponding amino acid residue was, therefore, involved into the active site if only one of its atoms was selected. Other default parameters were adopted in the SURFLEX-DOCK calculations. All calculations were performed on Silicon Graphics workstation (package version 6.9.1 on silicon graphics origin300 workstation with IRIX 6.5 operating system, San Francisco, CA, USA).

## 4. Conclusions

In summary, two series of sorafenib derivatives bearing sulfonylurea scaffold were designed and synthesized. All of the target compounds were evaluated the activity against four cancer cell lines and VEGFR2/KDR kinase. Six of the target compounds showed moderate activity and compounds **6c** and **6f** were better. The first series with no substitution in the phenoxy group showed more activity than the second series. Different substitutions of the aryl group affected the cytotoxicity of target compounds. Small halogen atom substitutions of the aryl group contributed to the activity of the first series, while there is no significant regularity of the second series. Although all of the target compounds showed less activity than the positive compounds, structure-activity relationships (SARs) and docking studies indicated that sulfonylurea unit is important to the activity of this kind of compounds. The results suggested that the sulfonylurea sorafenib analogs are worthy of further study. More compounds of sorafenib analogs bearing a sulfonylurea may be screened by replacing the aryl groups by heterocyclic rings in our further study.
